# Optimization of PETG 3D printing parameters for the design and development of biocompatible bone implants

**DOI:** 10.3389/fbioe.2025.1549191

**Published:** 2025-03-27

**Authors:** M. Moeen Sultan, Tauseef Aized, M. Farooq, Saqib Anwar, Naseer Ahmad, Ambreen Tauseef, Fahid Riaz

**Affiliations:** ^1^ Department of Mechanical Engineering, University of Engineering and Technology, Lahore, Pakistan; ^2^ Department of Mechanical Engineering, College of Engineering, Prince Mohammad Bin Fahd University, Khobar, Saudi Arabia; ^3^ Industrial Engineering Department, College of Engineering, King Saud University, Riyadh, Saudi Arabia; ^4^ Department of Physiology, CMH Medical College and Institute of Dentistry, Lahore, Pakistan; ^5^ Department of Mechanical Engineering, Abu Dhabi University, Abu Dhabi, United Arab Emirates

**Keywords:** additive manufacturing, 3D printing, ANOVA, optimization, cranial implant, polyethylene terephthalate glycol, RSM, CT scan

## Abstract

The search for suitable manufacturing methods and the selection of biocompatible material with good mechanical properties is still a major challenge in implant development. polyethylene terephthalate glycol (PETG) is a thermoplastic extensively utilized in biomedical applications, like tissue engineering, dental, scaffolds and surgery, because of its biocompatibility. Fused deposition modeling (FDM) is gaining importance in wide range of applications for developing custom shaped medical implants. This study aimed to fabricate a cranial implant using the optimized parameters of 3D printed PETG for good mechanical properties. The research investigates the optimization of key printing parameters like layer height, line width and print speed for PETG material by utilizing Box Behnken Design (BBD). Analysis suggests that the influential parameters of FDM are layer height and line width, which significantly influence tensile and compressive strength. The analysis of variance (ANOVA) showed that a layer height of 0.12 mm, line width of 0.77 mm and print speed of 25.75 mm/s indicated the increased value of tensile and compressive strength, i.e., 51.18 MPa and 52.33 MPa, respectively. The effectiveness of the RSM model was confirmed using the validation experiment, with errors less than 2%. Additionally, this study presents the process framework for the development of customized cranial implants by using computed tomography (CT) scan data of the patient. The 3D printed implant tested under uniaxial compressive load shows an average peak value of 1088 N. The goal of this research is to assist surgeons in overcoming clinical challenges faced while selecting materials and in-house production of patient-specific implants. A further evaluation of the presented technology is recommended for its potential use in clinical trials.

## Graphical Abstract

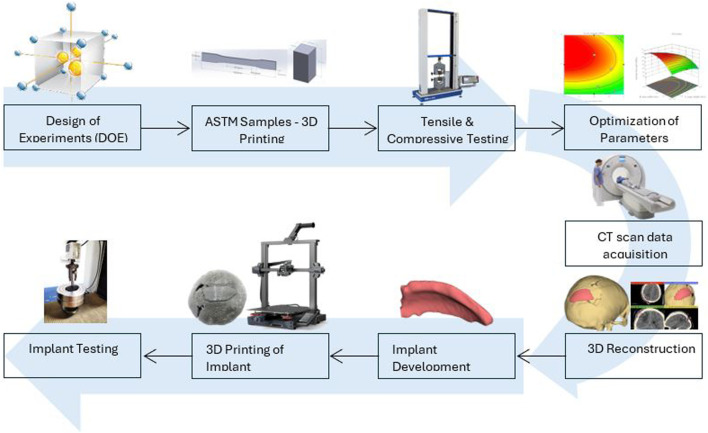

## Highlights

• The research identifies the optimized 3D printing parameters for PETG in the biomedical field by enhancing the tensile and compressive strength.
• Box Behnken design RSM is used to identify the ideal settings for layer height, line width and printing speed.
• Optimized values are used to 3D print customized cranial bone implants by using patient’s CT scan data, crucial for medical applications.

## 1 Introduction

Cranioplasty is the surgical procedure to reconstruct cranial defects resulting from accidental trauma injuries or the excision of brain tumors (Klammert et al., 2010; Lethaus et al., 2012; Shah et al., 2014). Damage in cranial bone exposes the brain to external forces which compromise the protection of the brain. When damaged, it needs to be reconstructed by using implants to restore the protective barrier and improve the aesthetics of the skull (Bonda et al., 2015; Piazza and Grady, 2017; Kaya et al., 2023). For surgeons, cranioplasty is a significant challenge regarding surgery time and achieving a precise implant fit. The common method for restoration of bone defects is bone grafting, which is suitable for smaller damage, but for larger and irregular bone defects, it becomes challenging and creates surgery related complications and increases the operation time (El Halabi et al., 2011; Lethaus et al., 2014; Joyce et al., 2023).

To resolve these clinical complexities, prefabricated implants of synthetic materials like metals, metal alloys, ceramics and thermoplastics are used by surgeons for cranioplasty (Zanotti et al., 2016; Msallem et al., 2017; Alkhaibary et al., 2020). Young’s modulus of commonly used metal implants like titanium and alloys is higher than human bone, which leads to stress shielding on the surrounding bone and contributes to a decline of bone density. Preferably, the mechanical properties of bone and implant should be matched for better integration and reduction in the risk of osteolysis (Huiskes et al., 1992; Bai et al., 2019). When these metal implants encounter variations in ambient temperature, they exhibit high thermal conductivity, causing discomfort and complications in patients (Alakhras and Alakhras, 2020; Li et al., 2024). Recent studies have shown that titanium may exhibit toxic reactions such as the corrosion or wear of titanium implants, which can cause small particles and ions to be released, accumulating in the surrounding tissues (Kim et al., 2019). This results in bone resorption caused by inflammatory responses, which lead to implant failure at the site (Albrektsson et al., 2018). However, it also becomes challenging to customize the autologous grafts and prefabricated implants, which cause misalignments and compromises the healing process of the patient (Alkhaibary et al., 2020; Sharma et al., 2020; Xu et al., 2022). These limitations piqued great interest among researchers in finding alternate materials for patient-specific implants (PSI).

In the recent decade, rapid development has been observed in computer-aided technologies for designing of cranial and dental implants and their fabrication to enhance surgical performance (Kim et al., 2018; Petersmann et al., 2020; Schweiger et al., 2021). Most significantly, using AM for developing orthopedic implants has been extensively researched to improve the alignment and functioning of the implant. Numerous studies have focused on creating complex anatomically shaped implants by acquiring CT scan of the patient and acquiring a three-dimensional model using design software (Wong, 2016; Modi and Sanadhya, 2018; Huang et al., 2022). The digital model in stereolithography (STL format) is then used to manufacture custom shaped implants using additive manufacturing techniques that could build complex geometrical shapes within hours (Tsouknidas et al., 2012; Moncayo-Matute et al., 2022). FDM is extremely popular among different AM techniques available due to its operational ease, intrinsic material flexibility and affordability (Mazzanti et al., 2019; Yadav et al., 2023). FDM technology utilizes a wide range of polymers and metallic alloys to develop orthopedic implants. Metal implants have several limitations due to mechanical mismatch and create hindrances in the natural tissue regeneration process, which may cause allergic reactions and inflammatory responses (Nagarajan and Reddy, 2009; Dang, 2014; Jamari et al., 2022). Polymers are crucial in the biomedical field because of their flexibility, biocompatibility, and cost-effectiveness when processed using FDM technology.

Previous studies revealed that the significant characteristics of implants made from polymers have advantages of chemical stability, hydrophilicity, surface charge and topography (Anderson, 1988; Franz et al., 2011; Zaveri et al., 2014; Corradetti, 2016; Yanez et al., 2017; Carnicer-Lombarte et al., 2021; Rausch et al., 2021). Polymers have certain limitations like biocompatibility, biodegradability and weak mechanical properties, and there are varying reports mentioning their suitability for medical applications (Kurowiak et al., 2023; Farjaminejad et al., 2024). Thermoplastic filaments of polyether ether ketone (PEEK), polyethylene terephthalate glycol (PETG) and polylactic acid (PLA) have good biocompatibility and can be utilized for medical purposes, especially in development of bones (Jamari et al., 2022). Some other FDM printable base materials are polymethyl methacrylate (PMMA), polyvinylidene fluoride (PVDF) and polypropylene (PP). All of the above-mentioned materials are approved under Food and Drug Administration (FDA) (Benyathiar et al., 2022). Biocompatibility is a crucial parameter for material selection which indicates the implant’s performance inside the body without any reaction to body fluids and inflammation (Ratner, 2015; Teo et al., 2016). PEEK is a high-performance thermoplastic used for applications that need high load bearing capabilities like orthopedic, dental and fixation of plates and screws (Panayotov et al., 2016). PEEK has low heat conductivity compared to metals but has limited potential in cellular behavior in the surrounding bones and produces an environment that favors cell death via apoptosis (Olivares-Navarrete et al., 2015; Phan et al., 2016; Zaman et al., 2019) used PLA and PETG to build aerospace parts, and the results show that PETG parts have better compressive strength than PLA parts. PETG presents a lower glass transition temperature (Tg) ranging from 70°C to 80°C. The lower thermal resistance of PETG makes it particularly suited for applications that do not require exposure to extreme temperatures and can be utilized in medical applications where temperature exposure is moderate.

Poor biocompatibility may result in inflammatory reactions and increase the risk of bacterial infections. PETG is a thermoplastic with good biocompatibility and is used in prosthetic, reconstruction surgeries, bone tissue engineering and provides an environment that supports enhanced cell attachment (Hassan et al., 2020). When PETG samples are tested in cell culture, they demonstrate notable cytocompatibility with bone marrow after 12 h of cultivation, making it particularly applicable for diverse medical practices like dental implants, scaffolds, bone implants and tissue engineering (Shilov et al., 2022). Similar results were observed for cytotoxicity in another study, where over 70% of exposed cells remained viable after 4, 8, and 12 days of incubation (Arany et al., 2021; Katschnig et al., 2020) successfully fabricated implant for a maxillofacial defect in an *ex-vitro* setting and suggested TPU/PETG implant as a promising solution for patients who face high-risk of implant rejection and are exposed to high-impact stress.

With advancements in FDM printing, the key challenge is adjusting printing parameters to achieve mechanical strength for surgical implants. PETG is an amorphous copolymer created by the addition of glycol during a polyethylene terephthalate (PET) synthesis (Vidakis et al., 2021). PETG is considered a better polymer than PET for 3D printing (Schneevogt et al., 2021). Apart from its excellent biocompatibility, it also exhibits good tensile strength, flexibility, and relatively strong interlayer bonding compared to other materials processed using FDM (Dolzyk and Jung, 2019; Bex et al., 2021). Many parameters like printing temperature, infill density, raster angle, infill pattern, layer height, printing speed, etc., could influence the product’s strength (Kafle et al., 2021). In FDM, the desired shape is accomplished by the relative movement of the printing head against heated platform using layer by layer method (Hossain et al., 2022).

(Lakshman et al., 2024) indicate an increase in tensile and compressive strength by rising nozzle temperature, bed temperature and infill density for PETG material. A reduction in strength was observed while increasing layer height and printing speed (Costanzo et al., 2020). claims that the welded strength, measured perpendicular to the print direction, decreases by increasing printing speed and by reduction in nozzle temperature (Mansour et al., 2018). analyze the modulus and hardness of the carbon-reinforced PETG samples and the result shows an increase of 30 and 27 percent, respectively, compared with simple PETG (Zarko et al., 2017). concluded that the elements printed at high print speed settings increase the porosity and voids, which results in a rougher surface and reduced details (Guessasma et al., 2019). determined tensile performance within the limited range of 210°C–250°C printing temperature and the result indicated that maximum tensile strength of PETG filament was observed at 250°C. When the temperature is below 230°C, an adhesion problem occurs between the print bed and the printed PETG sample.

(Hanon et al., 2019) observed that 0° raster angle results in an increased value of tensile strength and elongation of PETG material (Ozen et al., 2021). observed the impact of layer height of PETG as well as the overlap ratio and concluded that the mechanical strength was enhanced as the value of layer thickness was reduced and the overlap ratio was increased (Srinivasan et al., 2020). studied different infill patterns and reported that the grid pattern shows an increased value of tensile strength, i.e., 36.34 MPa for PETG material (Barrios and Romero, 2019). used Taguchi and ANOVA analysis to analyze the impact of process speed acceleration and flow rate on surface roughness for PETG and suggested that these parameters are the most influencial (Hsueh et al., 2021). revealed that enhancing the nozzle temperature from 225° to 245° increases strength and the increase in printing speed from 25–35 mm/s reduced the strength of FDM printed PETG and PLA parts (Dolzyk and Jung, 2019). tested the PETG samples for four different raster orientations and obtained the maximum value of tensile stress ranging from 41.58 MPa to 48.04 MPa, where the longitudinal specimen exhibits the maximum value (Kumaresan et al., 2023). assessed that for PETG samples increased value of tensile strength was accomplished by the use of a concentric pattern, while a triangular pattern enhanced the compressive strength. Furthermore, the increase in density from 25%–75% significantly increased the mechanical strength (Loskot et al., 2023). has found that voids and morphological defects occur as the printing speed exceeds 60 mm/s for PETG printed samples. Printing speed is crucial during 3D printing as it determines the speed with which the printing head travels. By increasing the printing speed, the print time is reduced, as it affects various properties due to over-extrusion or under-extrusion of material (Algarni and Ghazali, 2021; Ghodbane et al., 2019) observed that the primary factor in fused deposition modeling is the printing speed, as it influences the molecular orientation in the 3D printed sample. Molecular chain alignment is a key contributor that influences the mechanical properties (Hassan et al., 2021).

Various design of experiments (DOE) techniques like RSM, ANOVA, Taguchi and full factorial design are used to obtain the optimum value of 3D printing parameters (Alafaghani and Qattawi, 2018; Wu and Chiu, 2021; Gao et al., 2022) compared Taguchi and RSM, two widely used techniques for the optimization of printing parameters using PLA samples. Both techniques predict better results for tensile and compressive strength with minor prediction errors. RSM predicts higher optimum combinations compared to Taguchi. (Rashed et al., 2022). made a comparison between two techniques of design of experiments (DOE), i.e., Taguchi and full factorial design to analyze mechanical strength and surface roughness. Taguchi is observed as an efficient technique with some errors to optimize the parameters (Kechagias and Vidakis, 2022). optimized PA-12 material using BBD and full factorial design. The study proved that both approaches present same value of efficiency for quadratic regression modeling (Tontowi et al., 2017). optimizes the printing parameters of PLA against maximum tensile strength. In this study, Taguchi and RSM techniques were compared, and the optimum values obtained by RSM gave better print quality than those obtained by Taguchi.

Traditionally, cranial implants have been fabricated using additive manufacturing with titanium alloys and various other biomaterials, chosen based on the surgeon’s preferences considering the implant’s size, shape, and location. Limited literature is available on optimizing mechanical strength for PETG material using FDM, especially related to the application of bone implants, which acted as a driving force for this research. Tensile and compressive strength are crucial performance indicators while accessing the material, which indicates the maximum load it can bear before breaking (Khorasani et al., 2022). These properties are crucial while evaluating implants as several forces act due to the expansion or contraction of the surrounding tissues and external forces and impacts. Based on the presented literature, the FDM parameters, including layer thickness, line width and print speed, are selected to increase the tensile and compressive strength of PETG material. Line width is the ignored parameter and is usually adjusted to the default value depending on the nozzle size. This research may provide insight into the interaction of selected parameters to have a balance between fine layers and fast production. This study aims to achieive maximum tensile and compressive strength by identify the optimal values of FDM parameters using BBD as a technique of RSM. Furthermore, using patient’s CT scan data, the customized cranial implant was designed using our proposed workflow, resulting in a better fit for the skull. PETG is a biomaterial, and the mechanical strength of customized cranial implants developed by FDM using PETG needs further research to be introduced in clinical trials. The rest of the paper comprises Sect. 2, which presents “Materials and Methods” explaining experimental setup and testing procedures. Section 3 presents analysis of the research and discusses the optimization of FDM parameters of PETG. Finally, the steps involved in designing cranial implants using CT scan data of the patient and its testing are presented in Section 4. Section 5 presents the conclusion.

## 2 Experimental methodology

### 2.1 Material and FDM

Sky Fila brand Polyethylene Terephthalate Glycol (PETG) filament with 1.75 mm diameter was used, purchased from Sky Heights, Pakistan, as a feedstock to manufacture samples and the material properties are listed in Table 1 (Skylabs, 2025). PETG has been selected for printing human bone implants because of its mechanical strength, biocompatibility, durability, high impact and chemical resistance. The FDM-based 3D printer Creality Ender 3-S1 (Shenzen, China) was utilized for the printing of experimental samples. In FDM, the thermoplastic filament is melted using a hot extruder and the fused material is used to build the 3D object (Wickramasinghe et al., 2020; Rahmatabadi et al., 2023). The printer has several valuable features like auto bed leveling and print resume on power loss, having single nozzle of 0.4 mm diameter installed. Different settings can be achieved to speed up the prints with low power consumption (Manford et al., 2023).

**TABLE 1:** Properties of PETG filament.

Properties	Units	PETG
Tensile strength	MPa	41.5–44.5
Impact strength	KJ/m^2^	5–6
Elongation at break	%	10–12
Flexural Strength	MPa	64.5–66
Printing Temperature	^o^C	220–240
Material density	g/cm^3^	1.27–1.28

### 2.2 Experimental design matrix

**FIGURE A1:**
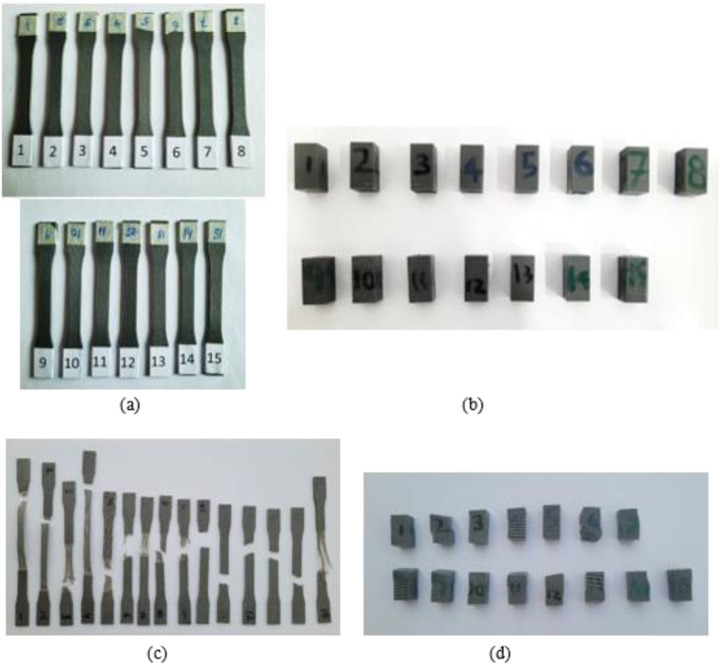
**(a)** FDM printed tensile samples. **(b)** FDM printed compression samples. **(c)** Samples after tensile testing. **(d)** Samples after compressive testing.

Response surface methodology (RSM) is a collection of statistical methods that are often utilized to analyze problems for the optimization of the process variables (Equbal et al., 2021). The study utilized Box Behnken design (BBD) technique of RSM for investigating the effect of FDM parameters on tensile and compressive strength. BBD was chosen as it reduces the number of runs and can be used for large number of factors in one process (Box and Behnken, 1960). The experimental design was set in a systematic order to perform the experiments and analyze the response outcomes using Design Expert software (version 12). The variables identified for this research were line width, print speed and layer height. Other parameters, like nozzle temperature, infill density, platform temperature, raster angle and nozzle size, are kept constant during the experimentation, tabulated in Table 2. However, the choice of selected input parameters for optimization and ranges of factors in three distinct levels are listed in Table 3. The output responses were assessed for tensile and compressive strength. Both are fundamental properties for the design of cranial implants, as the performance of implants is dependent on how well the material resists the applied stress. A total of 15 experiments were formulated by Design Expert Software using BBD and respective test samples were fabricated on 3D printer using values from each run. The test specimens used for mechanical testing are presented in Figures A1A, B. The samples were tested on UTM for each experimental trial, and the output response was recorded. Figures A1C, D shows the tensile and compressive samples after testing.

**TABLE 2:** Fixed printing process parameters for FDM printer.

Printing parameters	Units	Values
Extrusion Temperature	°C	240
Infill Pattern	--	Lines
Platform Temperature	°C	90
Infill Density	%	100
Nozzle Size	mm	0.4
Raster Angle	Degree	0

**TABLE 3:** Variable FDM Printing Parameters and their stage values.

Parameters	Units	Stage 1	Stage 2	Stage 3
Layer Height [A]	mm	0.1	0.2	0.3
Line Width [B]	mm	0.4	0.6	0.8
Printing Speed [C]	mm/s	25	35	45

To develop mathematical models for the experimental data of tensile and compressive strength, the quadratic Equation 1 is used. This mathematical quadratic model of the second order polynomial represents the relation between input variables and output responses

$$Y=\beta_{0}+\sum_{i=1}^{n}\beta i{\;x}_{i}+\sum_{i=1}^{n}\beta \text{ii}{{\;x}_{i}}^{2}+\sum_{i}\sum_{j}\beta \text{ij}{\;x}_{i}{\;x}_{j}+e$$ (1)

Where β_0_ is constant, β_i_ represents the constant of the linear term, β_ii_ refers to the quadratic coefficient, β_ij_ represents the co-efficient of interaction terms, e represents an experimental error, $x_{i}{\;\&\;x}_{j}$ are independent variables and n is the number of factors used for experimentation.

### 2.3 Specimen fabrication and testing

**FIGURE 1:**
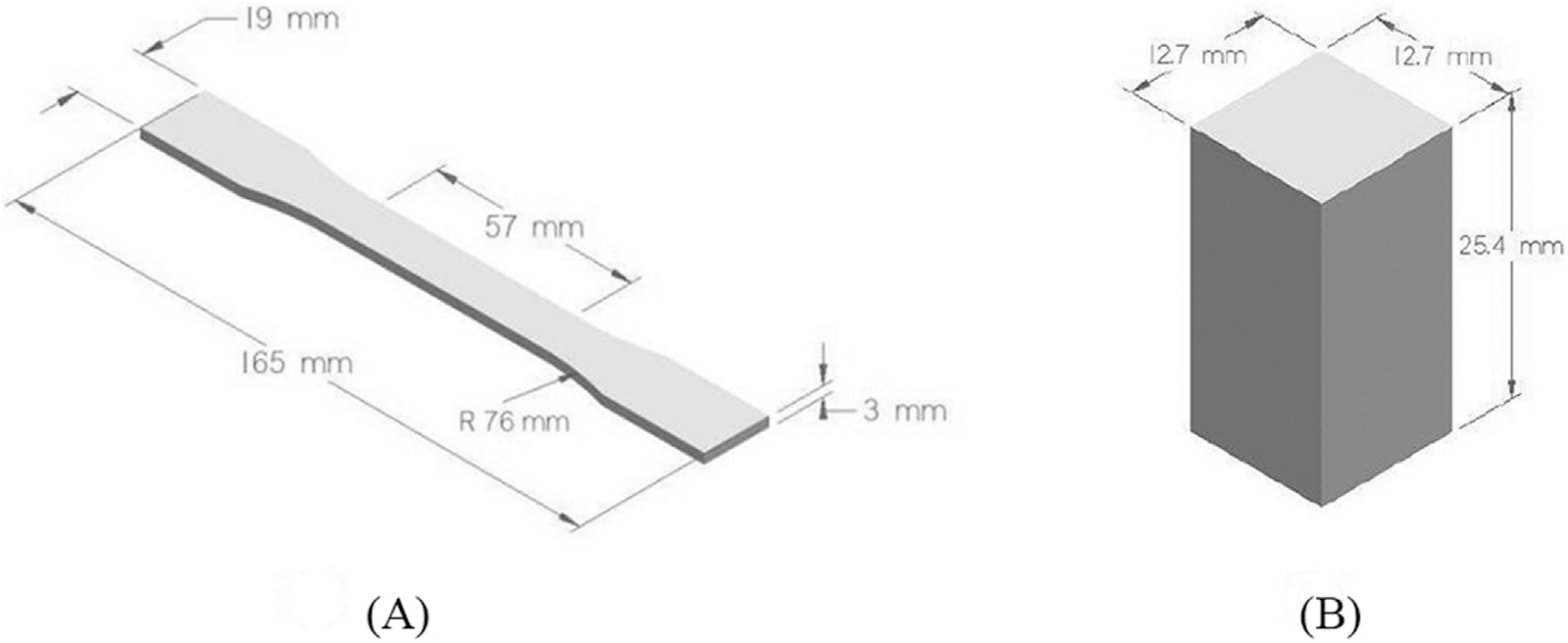
**(A)** Model of tensile test sample **(B)** 3D model of the compressive specimen.

PETG material undergoes tensile and compressive tests to evaluate the influence of printing parameters on mechanical strength. To investigate the effect of printing parameters on mechanical strength, the PETG material should undergo tensile and compressive tests. To find the optimal values of FDM parameters, the preparation of standard test specimens was required. The samples needed for strength optimization were designed based on American Society of Testing and Materials (ASTM) standards, ASTM–D 638 (Type-I) for tensile testing and ASTM - D 695 for compressive testing (Gotkhindikar et al., 2023). The models were developed in solid modeling software CREO and stored as STL files. Figure 1 illustrates the dimensions of tensile and compressive test samples.

The variation in process parameters was achieved using the open-source software Ultimaker Cura, which slices the input STL file and generates G-codes. These G-codes were fed as an input to FDM printer to develop the test specimens. The values of printing parameters in the fabrication process affect the mechanical strength of the finished part differently.

### 2.4 Sample testing procedure

**FIGURE 2:**
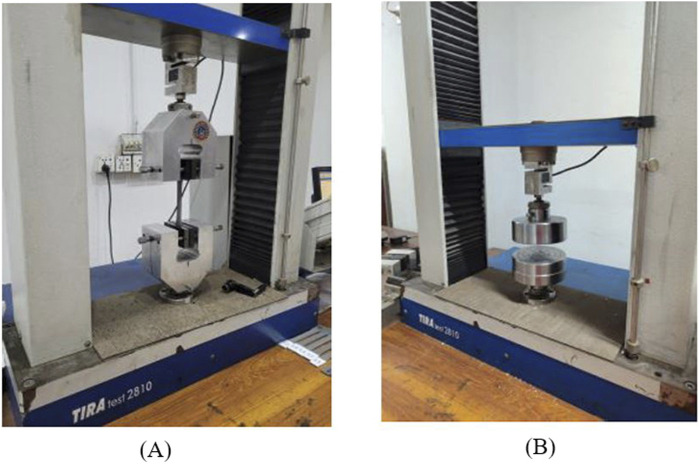
**(A)** TIRA test 2810 UTM tensile **(B)** TIRA test 2810 UTM compression.

To examine the mechanical properties (TS and CS) of PETG, the FDM specimens were tested by utilizing Universal Testing Machine (UTM). For accurate testing, UTM machine should have appropriate load cells and displacement sensors that can measure stress and deformations of samples. The tensile and compressive testing of samples was performed using TIRA test 2,810 (Germany) UTM, containing a load cell of 10 kN at room temperature to measure the force on specimens, as illustrated in Figures 2A, B. To ensure accuracy and repeatability, the samples of tensile and compressive strength were subjected to three repetitions to obtain an average value for the consistency of the results. The crosshead speed of 5 mm/s was used to adjust the speed of loading or deformation applied to the specimen.

### 2.5 ANOVA analysis

The ANOVA (Analysis of Variance) is a mathematical tool to assess the input parameters that substantially influence the output response by fitting the full quadratic model (Gao et al., 2022). This test simultaneously facilitates a concurrent evaluation of several groups for the most significant parameter influencing the mechanical properties of teste samples. Design Expert software was used to conduct ANOVA analysis and fit a model based on the response surface using linear, quadratic and two-way interactions of three input parameters. The results are analyzed below, and the interpretation of data for both TS and CS is graphically represented using the main plots.

## 3 Results and discussion

This study explored the influence of FDM parameters on the tensile and compressive strength of PETG material to develop cranial bone implants. There were 15 test specimens on which tensile and compressive tests were conducted separately. Regression model equations are established using RSM technique in Design-Expert software. These equations helped predict the responses of TS and CS for the printed specimens. The experimental design followed for PETG samples as well as the corresponding values of both the actual data and the predicted values, as presented in Table 4. The responses were analyzed using ANOVA, and the significance of the model was tested to achieve the desired output.

**TABLE 4:** Comparison between experimental and predicted values.

Run #	Layer height (A)	Line width (B)	Print speed (C)	Tensile strength –TS (MPa)			Compressive strength - CS (MPa)		
				Experimental	Predicted	Error %	Experimental	Predicted	Error %
1	0.3	0.6	45	47.58	47.37	0.2063	45.84	46.03	−0.1925
2	0.2	0.4	25	48.37	48.22	0.1462	48.49	48.63	−0.1375
3	0.1	0.6	25	51.16	51.37	−0.2062	49.86	49.67	0.1925
4	0.3	0.4	35	45.73	45.83	−0.1025	47.07	46.95	0.12
5	0.1	0.4	35	47.71	47.65	0.06	48.16	48.22	−0.055
6	0.2	0.6	35	49.46	49.66	−0.1967	48.23	48.33	−0.0967
7	0.3	0.8	35	46.52	46.58	−0.06	48.44	48.39	0.055
8	0.1	0.6	45	50.98	50.94	0.0437	48.33	48.35	−0.0175
9	0.2	0.4	45	47.15	47.25	−0.1038	48.42	48.35	0.0725
10	0.2	0.8	45	48.44	48.59	−0.1462	50.28	50.14	0.1375
11	0.2	0.8	25	50.28	50.18	0.1038	52.08	52.15	−0.0725
12	0.2	0.6	35	49.79	49.66	0.1333	48.54	48.33	0.2133
13	0.2	0.6	35	49.72	49.66	0.0633	48.21	48.33	−0.1167
14	0.3	0.6	25	49.46	49.5	−0.0437	47.02	47	0.0175
15	0.1	0.8	35	50.29	50.19	0.1025	51.98	52.1	−0.12

### 3.1 ANOVA analysis for TS

ANOVA results in Table 5 provide valuable insight regarding tensile strength against the input parameters of layer height (A), line width (B) and print speed (C). For tensile strength (TS) of the developed PETG sample, the fisher value ‘F' of 86.12 implies the significance of the model with a ‘P' value < 0.0001 (Hong et al., 2014). This shows the validity of the model having only 0.01% chance of noise generation. The factors with P values less than 0.05 substantially influence the RSM model. In this model, the individual parameters (A, B, C), interaction parameters (AB, AC) and quadratic values (A^2^, B^2^, C^2^) are significant for tensile strength of PETG. Statistical data for tensile strength yielded an *R* ^2^ value of 0.9936, indicating a higher level of reliability in modeled data due to the value closer to 1. The adjusted *R* ^2^ of 0.9821 is also close to 1, which shows that *R* ^2^ and adjusted *R* ^2^ are in reasonable agreement. The predicted *R* ^2^ value of 0.9195 represents that the model has high predictive accuracy. The adequate precision of 30.8134 (S/N > 4) indicates a sufficient signal and suggests that the model can be used for optimization. The standard deviation value of 0.22 represents that the investigational outcomes are closely aligned with the predicted model. This analysis indicated that layer height and line width are significant variables as compared to print speed.

**TABLE 5:** ANOVA for tensile strength.

Source	SS	DOF	MS	F-value	p-value
Model	37.5	9	4.17	86.12	<0.0001
A-Layer height	14.72	1	14.72	304.17	<0.0001
B-Line width	5.4	1	5.4	111.53	0.0001
C-Print speed	3.28	1	3.28	67.73	0.0004
AB	0.801	1	0.801	16.56	0.0096
AC	0.7225	1	0.7225	14.93	0.0118
BC	0.0961	1	0.0961	1.99	0.2178
A^2^	0.6814	1	0.6814	14.08	0.0133
B^2^	10.23	1	10.23	211.47	<0.0001
C^2^	1.19	1	1.19	24.62	0.0042
Residual	0.2419	5	0.0484		
Lack of Fit	0.1814	3	0.0605	2	0.3504
Pure Error	0.0605	2	0.0302		
Cor Total	37.74	14			

The regression Equation 2 in terms of coded factors was derived for the output response of tensile strength (TS) and the three input parameters layer height (A), line width (B) and print speed (C) using design expert software analysis. The prediction of tensile strength in Equation 2 represents the nonlinear relationship between input parameters. The comparison of coefficients in the equation gives valuable information about the influence of each input parameter on output response. A positive coefficient value represents that increasing the input factor results in the increase of output response. In contrast, a negative coefficient value shows that the increase in the input factor decreases output response.

$$\text{TS}=+49.66-1.36\;A+0.8212\;B-0.64\;C-0.4475\text{ AB}-0.425\text{ AC }–\;0.155\text{ BC}-0.4296\;A^{2}-1.66\;B^{2}+0.5679\;C^{2}$$ (2)

**FIGURE 3:**
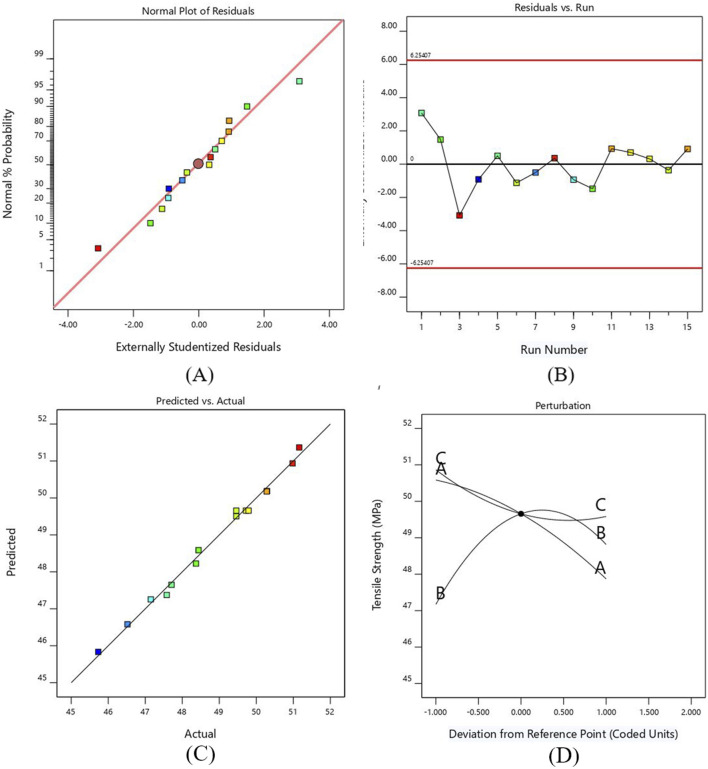
**(A)** Probability versus plot of residuals for TS **(B)** Normal plot of residuals with run number for TS **(C)** Plot between actual and predicted values for TS **(D)** Perturbation plot for TS.

In Figure 3A, the normal probability graph of residuals showed that the residuals lie approximately along a straight line for tensile strength. This close alignment of residual points suggests normal data distribution and represents an adequate model. Figure 3B illustrates the plot for normal probability residuals versus the runs. The sequence of experimental runs is plotted as small squares for each data point. The graph indicates the data points are randomly scattered without exceeding the upper and lower limit lines. Figure 3C shows relation of the predicted values of tensile strength from RSM model and the actual value from the experiments. The result of the graph shows that the predicted and experimental data closely align with the straight line, which implies that the suggested model is adequate and reliable.

A perturbation plot was mapped to exhibit the effect of printing parameters on the TS of PETG (see Figure 3D. The response can be plotted by varying one parameter across its entire range while keeping the other parameter constant. Parameters A and B have more significant deviations from the center reference point than parameter C. The graph shows that the TS of the developed PETG specimens decreases by increasing layer height and printing speed due to the development of weaker interlayer bonding (Raja et al., 2024). As the line width increases, the value of tensile strength increases to a specific limit and then decreases. This decrease in value occurs due to premature failure of samples caused by the formation of stress risers. This relationship indicates that balance is needed while increasing the line width, as excessively wide lines can create irregular geometry and negatively impact the strength due to the creation of typically weak points, leading to premature failure of samples (Kim et al., 2020).

### 3.2 ANOVA analysis for CS

Compressive strength (CS) is a critical response in evaluating the mechanical strength of FDM printed specimens. Compressive strength defines the specimen’s ability to withstand the force when it is under compression load. ANOVA analysis was performed in this section to evaluate the influence of input parameters, layer height (A), line width (B), and printing speed (C) on the CS of 3D printed samples, as presented in Table 6. The Fisher value “F” was 99.52, which indicated the significance of the model.

**TABLE 6:** ANOVA for compressive strength.

Source	SS	DOF	MS	F-value	p-value
Model	40.55	9	4.51	99.52	<0.0001
A-Layer height	12.4	1	12.4	273.9	<0.0001
B-Line width	14.15	1	14.15	312.57	<0.0001
C-Print speed	2.62	1	2.62	57.92	0.0006
AB	1.5	1	1.5	33.15	0.0022
AC	0.0306	1	0.0306	0.6764	0.4482
BC	0.7482	1	0.7482	16.53	0.0097
A^2^	1.99	1	1.99	44.01	0.0012
B^2^	6.44	1	6.44	142.19	<0.0001
C^2^	0.1072	1	0.1072	2.37	0.1844
Residual	0.2264	5	0.0453		
Lack of Fit	0.1579	3	0.0526	1.54	0.4174
Pure Error	0.0685	2	0.0342		
Cor Total	40.78	14			

The “P” value <0.05 implies that the input parameter has a major influence on the developed model. For the current model, A, B, C, AB, BC, A^2^, B^2^ are influential for compressive strength. The obtained values of *R* ^2^ is 0.9944, predicted *R* ^2^ is 0.9845 and adjusted R2 is 0.9343. As these values are close to 1, it shows that the suggested model performs well and will be a better fit for the prediction of output response. The adequate precision is 35.227 which is much greater than four and indicates an adequate signal. The smaller value of standard deviation, i.e. 0.213, indicates that the values of experimental data and predicted model are close to each other. In the case of compressive strength, line width is considered the most significant and print speed as the least significant variable in comparison with other parameters. The regression Equation 3 for compressive strength (CS) can be utilized for the generation of predicted response against the input variables. By comparing the coefficients of this coded Equation, it is helpful to identify which input factor has greater impact on the output response.

$$\text{CS}=+48.33-1.25A+1.33B-0.5725C-0.6125\text{AB}+0.0875\text{ AC}-0.4325\text{BC }–\;0.7346\;A^{2}+1.32\;B^{2}+0.1704\;C^{2}$$ (3)

**FIGURE 4:**
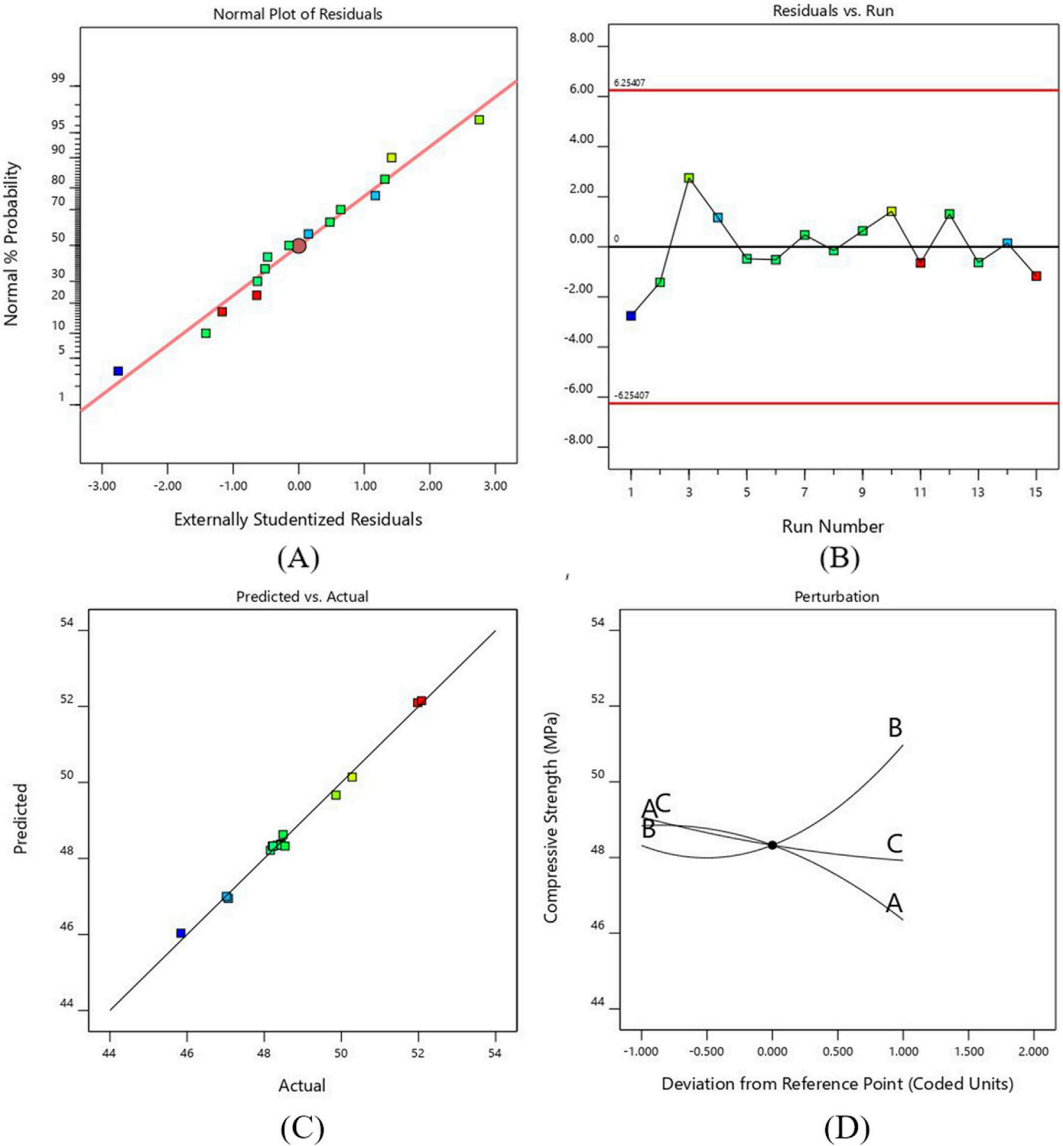
**(A)** Probability versus plot of residuals for CS **(B)** Normal plot of residuals with run number for CS **(C)** Plot between actual and predicted values for CS **(D)** Perturbation plot for CS.

The normal probability plot in Figure 4A. Represents that the residuals are normally distributed along a straight line, which shows that an adequate model is formed for compressive strength. The plot between the runs of compressive strength and externally studentized residuals is shown in Figure 4B. The residuals are randomly scattered around the zero line and lie within the expected upper and lower limits, which show the adequacy of ANOVA results.

Figure 4C shows that the actual compressive strength values obtained experimentally were close to the estimated values of regression polynomial model. The graph shows the accuracy of the predicted model as all the points align exactly with the observed data.

Figure 4D shows the perturbation plot for the compressive strength against three input parameters. The reference point was set in the center of each factor as 0.2 mm, 0.6 mm and 35 mm for layer height, line width and print speed, respectively. The graph shows how the change in each parameter affects compressive strength. The compressive strength increases as we increase the line width due to filling gaps by the wider lines, which requires more force to compress the samples. On the other hand, the value of compressive strength reduces as the layer height and printing speed increase. The decrease in layer height and print speed provides maximum area and time for bonding, resulting in stronger adhesion and increasing compressive strength (Almansoori and Pervaiz, 2023). However, the results obtained in this study indicate that higher print speeds reduce compressive strength due to insufficient adhesion between layers, leading to defects (Lakshman et al., 2024; Subramani et al., 2024).

### 3.3 Analysis of contour plot and interaction for tensile strength

**FIGURE 5:**
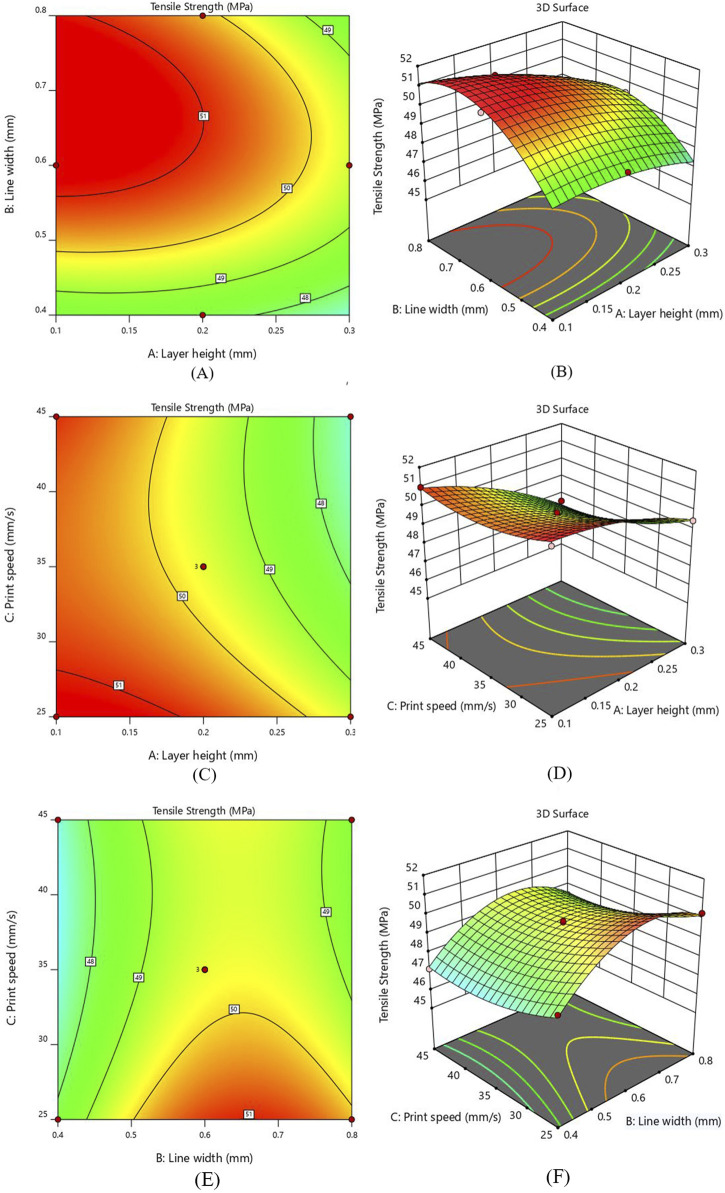
Interaction response for TS **(A)** layer height and line width - Contour plot **(B)** layer height and line width - 3D Surface plot **(C)** layer height and print speed - Contour plot **(D)** layer height and print speed - 3D Surface plot **(E)** line width and print speed - Contour plot **(F)** line width and print speed - 3D Surface plot.

Figures 5A, B represents the 3D RSM curve and 2D contour interaction between two input parameters of layer height (A) and line width (B) for TS. In this interaction study, a 25 mm/s print speed was adopted. The 3D RSM curve in Figure 5B represents that the effect of the AB interaction is not linear. The contour plot in Figure 5A represents the minimum and maximum values of resulting tensile strength plotted against the input parameters. The red-colored regions are the areas where the effect of the input parameters is maximum, and the green-colored areas have minimum response values.

Analyzing the contour plot in Figure 5A reveals that the TS shows a decreasing behavior with the increase in layer height while an increasing trend was observed as the line width increases, keeping print speed constant at 25 mm/sec. It was also observed that 0.1 mm layer height and 0.6 mm line width gave higher TS (51.36 MPa) as compared to the TS (47.35 MPa) of specimens printed with 0.3 mm layer height and 0.4 mm line width. Keeping the layer height constant and selecting a higher value of line width increased the tensile strength to a definite level and then slightly decreased. A decrease in layer height from 0.3mm to 0.1 mm at higher line width values increased the tensile strength. In Figure 5B, the 3D surface plot between AB indicated that the line width significantly increased TS, while the layer height was considered the least influential parameter. It has been determined that a lower layer thickness value and higher line width should be used to enhance the interlayer bonding, leading to more substantial 3D-printed parts (Almansoori and Pervaiz, 2023).

Figures 5C, D illustrates the contour and 3D surface plots for the interaction of layer height (A) and print speed (C). The value of line width was kept constant at 0.6 mm. The 3D plot indicates that the rise in layer height exhibits a decline in tensile strength irrespective of printing speed values. At a print speed of 25 mm/s, a reduction layer height from 0.3 mm to 0.1 mm results in an increase of TS from 49.50 MPa to 51.36 MPa, making a 3.8% improvement. Similarly, at 35 mm/s print speed, a 5.7% rise was observed in TS from 47.87 MPa to 50.58 MPa, as the value of layer height was reduced from 0.3 mm to 0.1 mm. Considering 45 mm/s print speed, a substantial increase of 7.53% of TS was observed with the reduction of layer height from 0.3 mm to 0.1 mm. These findings indicate that layer height significantly affects the tensile strength while print speed has the least influence. The lower layer height value and range of printing speed between 25mm and 45 mm give the highest performance.

As represented in Figures 5E, F, the peak value of tensile strength appears near the greater line width value and a smaller value of printing speed. The value of layer height was fixed at 0.2 mm for this interaction. It becomes apparent from the 3 days surface plot that printing speed has a minor effect, while an increase in line width significantly elevates the tensile strength (see Figure 5F). At 25 mm/s print speed, as the line width value increases from 0.4 mm to 0.8mm, a substantial rise in tensile strength was observed from 48.37 MPa to 50.28 MPa. At 45 mm/s print speed, the TS increases from 47.15 MPa to 48.44 MPa with the increment in line width. This validates a clear correlation between increased line width and enhanced tensile strength. The contour plot in Figure 5E shows that higher value of TS can be obtained when the print speed is sufficiently low and line width lies between 0.6 mm and 0.8mm, while the lowest tensile strength occurs when the printing speed is at its highest. Furthermore, it is observed that the interaction between line width and printing speed exerts a less pronounced influence on tensile strength as compared to other interaction effects based on the “F” value.

### 3.4 Analysis of contour plot and interaction for compressive strength

**FIGURE 6:**
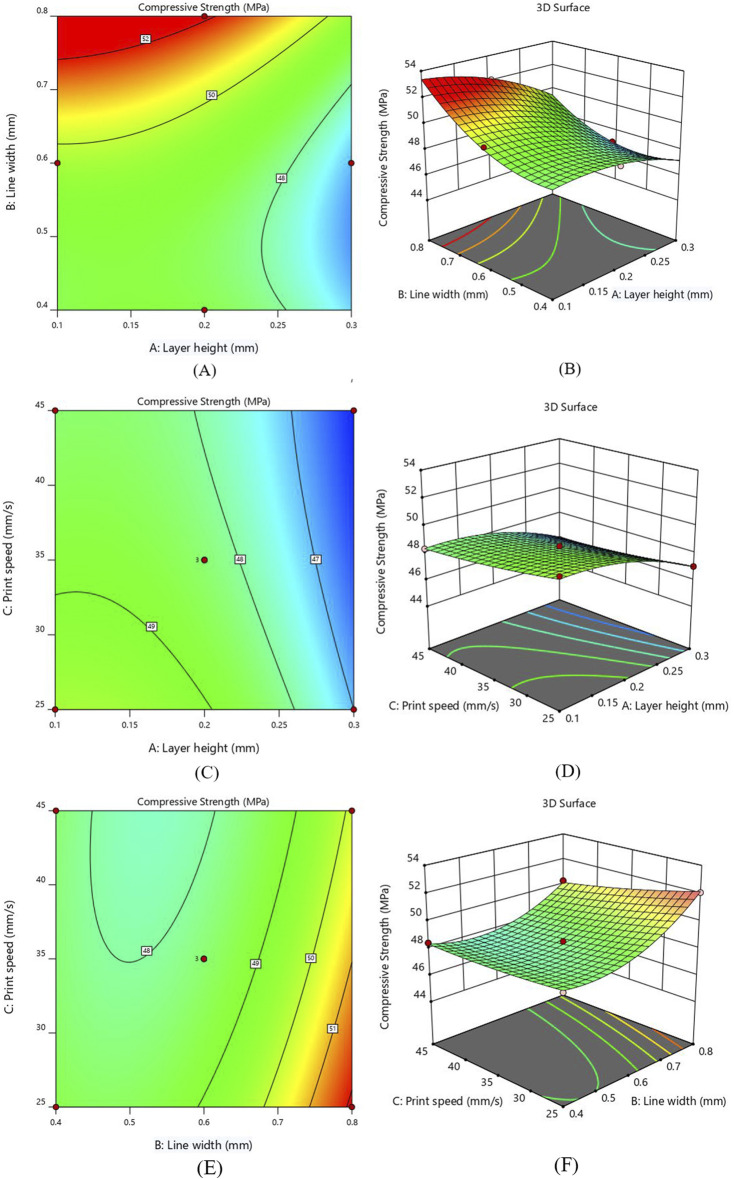
Interaction response for CS **(A)** layer height and line width - Contour plot **(B)** layer height and line width - 3D Surface plot **(C)** layer height and print speed - Contour plot **(D)** layer height and print speed - 3D Surface plot **(E)** line width and print speed - Contour plot **(F)** line width and print speed - 3D Surface plot Figure. Interaction of layer height and print speed for CS **(C)** Contour plot **(D)** 3D Surface plot.

Figures 6A, B illustrates the interaction of layer height (A) and line width (B) on the compressive strength (CS). The printing speed was kept constant at 35 mm/sec. The CS of the printed samples shows an increasing behavior as the value of line width rises from 0.4 mm to 0.8 mm, indicating the maximum CS value of 52.08 MPa. Similarly, a reduction in compressive strength was observed as the value of layer height increased from 0.1 mm to 0.3 mm and the line width decreased from 0.8 mm to 0.4 mm. The 3D RSM plot Figure 6B indicates that line width substantially influences the CS of the 3D printed sample. It is inferred that the 0.8 mm line width is the most suitable value for increasing compressive strength. Additionally, the decrease in layer height and increase in line width results in increased value of CS. Similar behavior was observed in previous studies in which compressive strength tends to increase as the layer height decreases due to stronger interlayer bonding (Chacón et al., 2017; Nyabadza et al., 2024).

The interaction effect for compressive strength between layer height (A) and print speed (C) are represented as 2D contour and 3D surface plots in Figures 6C, D. Notably, the compressive strength indicates an increasing behavior by decreasing layer thickness, as smaller layer height reduces the voids and allows for better adhesion between layers; hence, the chance of defects is minimized (Costanzo et al., 2020; Ozen et al., 2021). Figure 6D shows that the print speed does not significantly affect the value of CS. This suggests that utilizing fast print speed can be a viable option by not compromising compressive strength much, resulting in substantial savings in both time and energy. The inverse interaction effect between layer height and print speed was observed, indicating a reduction in compressive strength by increasing the value of both parameters. Furthermore, it was observed that the interaction of the A and C parameters has the least influence on CS.

Figures 6E, F illustrates the interaction between line width (B) and print speed (C) by keeping layer height constant at 0.2 mm. As illustrated in contour plot Figure 6E, the higher CS was obtained by reducing the value of print speed. The CS of the printed samples shows a rising behavior by increasing the line width due to the reduced number of voids (Patel et al., 2024). The contour and surface plots show that the line width significantly impacts compressive strength as compared to print speed, with the maximum value observed near 0.8 mm line width. A greater line width and a lower printing speed increase the contact area and bonding strength between layers, hence increasing the compressive strength.

### 3.5 Optimization of RSM model and validation

In this section, the optimization of independent variables is achieved through the desirability function to determine the best outcome. The desirability function ranges from 0 to 1, where 0 indicates an undesirable response value and one indicates the desired goal (Lee et al., 2018). The details of the minimum and maximum ranges of input parameters with the specific goals for each factor are indicated in Table 7. The numerical optimization goal was to maximize the TS and CS of the PETG samples. The desirability function for this optimization is 1.

**TABLE 7:** Optimization criteria for process parameters.

Sr No.	Name	Units	Goal	Lower limit	Upper limit	Importance
1	A: Layer height	mm	In range	0.1	0.3	3
2	B: Line width	mm	In range	0.4	0.8	3
3	C: Print speed	mm/sec	In range	25	45	3
4	Tensile Strength	MPa	Maximize	45.73	51.16	3
5	Compressive Strength	MPa	Maximize	44.32	52.67	3

**FIGURE 7:**
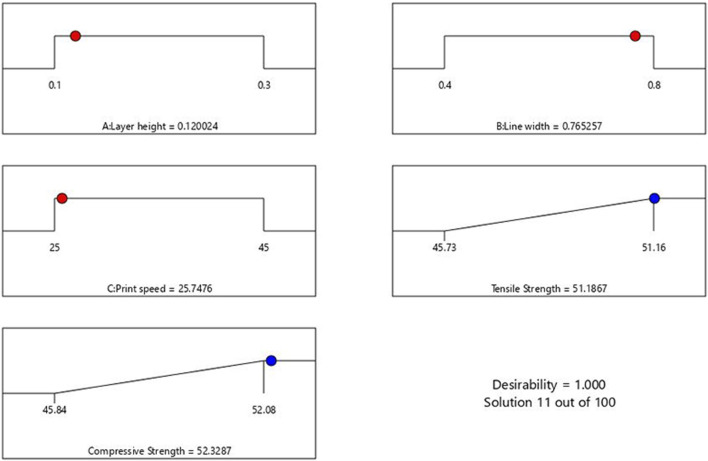
Ramp plot of optimized parameters.

**FIGURE 8:**
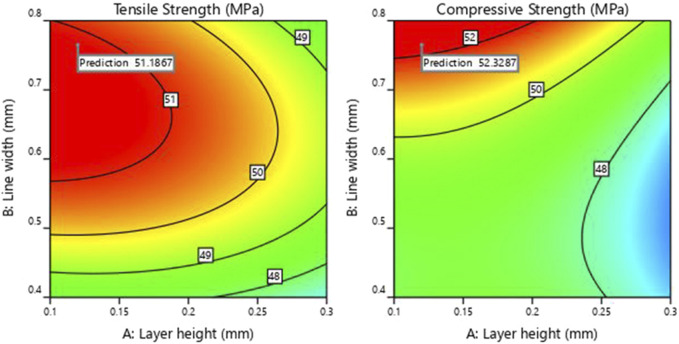
Contour of tensile and compressive strength.

As observed from Figure 7, the ramp plot shows the solution model of optimized parameters and the corresponding response. The optimized conditions for maximizing the tensile and compressive strength were obtained when the input parameters of layer height, line width and print speed were selected at 0.12 mm, 0.76 mm and 25.7 mm/s, respectively. The predicted tensile and compressive strength values obtained were 51.18 MPa and 52.33 MPa, respectively. The contour plot for the tensile and compressive strength of the samples generated against layer height and line width by keeping the printing speed at 25.7 mm/s, is shown in Figure 8.

**FIGURE 9:**
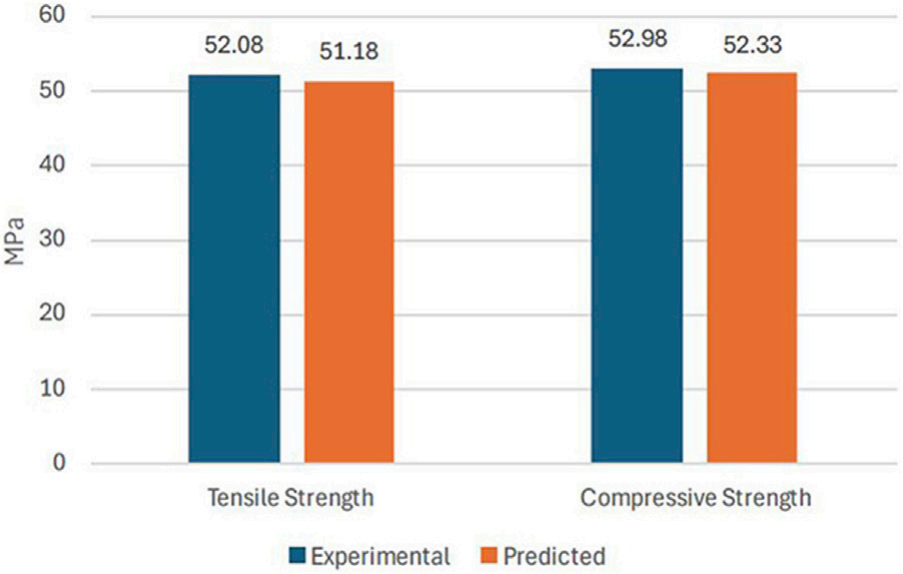
Validation of results.

**FIGURE 10:**
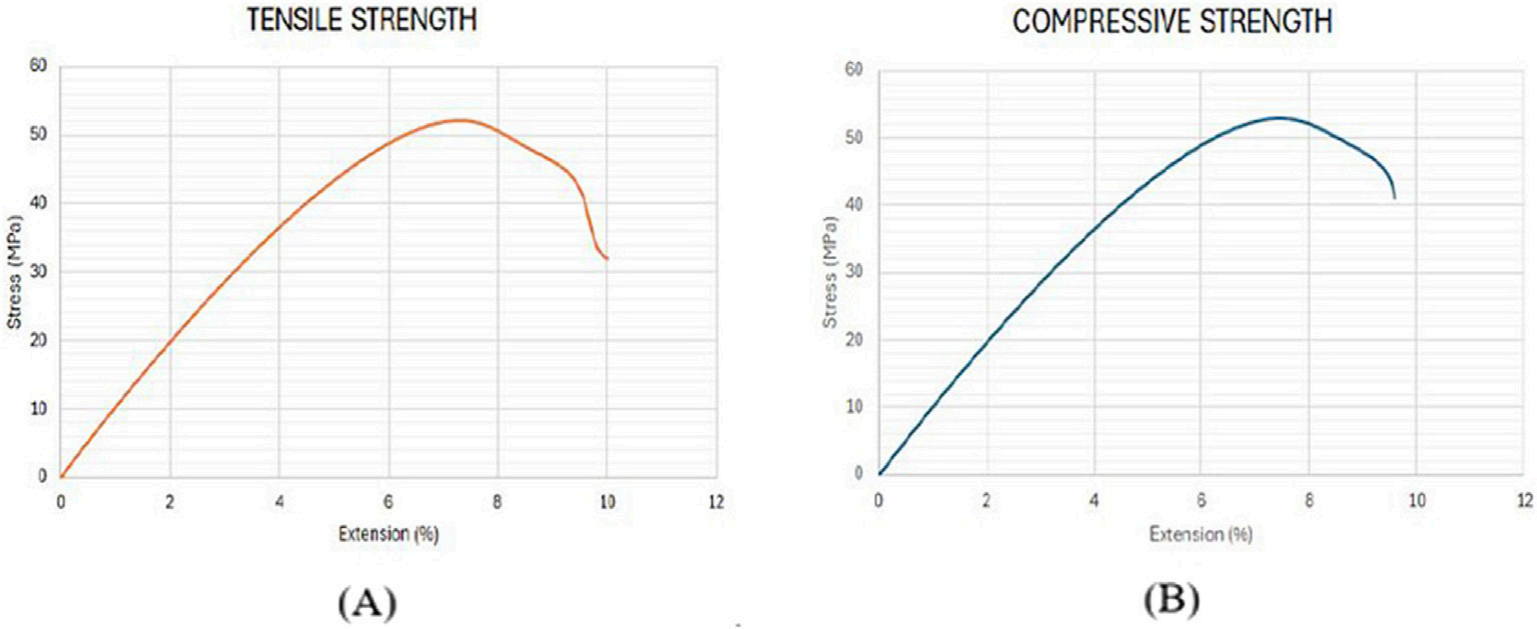
Stress-strain curve for validation samples. **(A)** Tensile Strength **(B)** Compressive strength.

Using the optimized values of FDM parameters, the samples were fabricated and tested to validate the predicted results of the RSM model. Figure 9 compares the experimental and the predicted values for tensile and compressive strength with an error of less than 2%. This confirms the predictability of the developed model using RSM, based on Box Behnken’s design. Figures 10A, B shows the experimental stress-strain graph for tensile and compressive strength, indicating maximum stress values of 52.08 MPa and 52.98 MPa, respectively. These results verify that the developed RSM model accurately predicts the optimal conditions.

## 4 Application in manufacturing cranial implant

Fused deposition modeling extensively used in reconstructing orthopedic, trauma, and dental implants in the past few years. Previously, manual shaping and conventional manufacturing methods have been utilized in the construction of cranial implants. Due to the growing demand for cranioplasty, the recent trend towards FDM has a remarkable application in developing cranial implants with materials that are lightweight and have high-strength characteristics. Based on proper planning, FDM can fabricate complex anatomical parts by acquiring CT scan images for better visualization (Abdulhameed et al., 2019). In the current study, a cranial implant has been developed using CT scan data of a patient who had a trauma head injury caused by a severe road accident. Now, considering optimized values of parameters for PETG material, the cranial bone implant will be fabricated using FDM technology. A compression test will then be used to investigate the mechanical behavior of the manufactured cranial implant.

**FIGURE 11:**
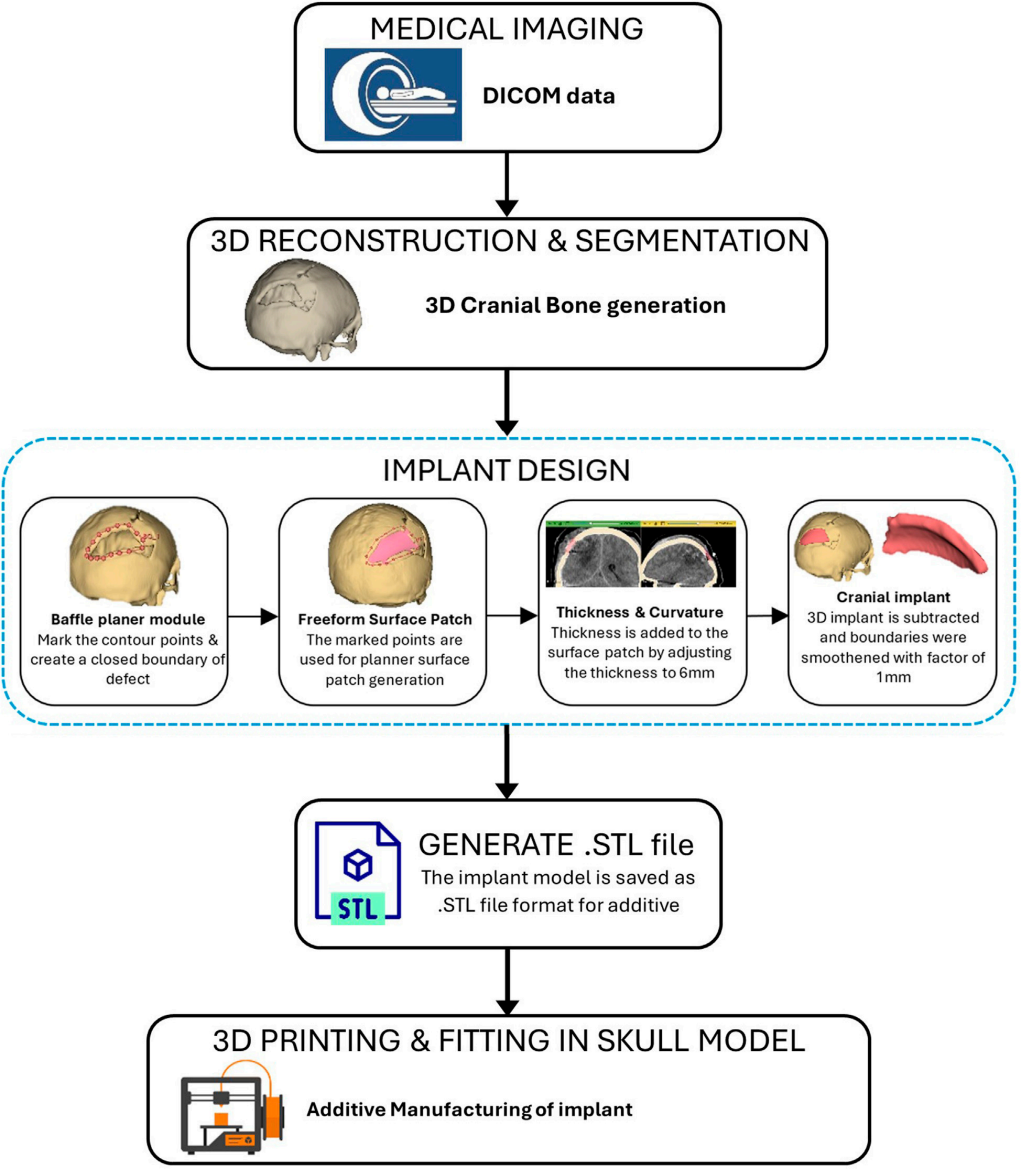
Workflow for the development of bone implant from CT scan.

As represented in Figure 11, the procedure involved in developing cranial implants from CT scan data involves four major steps: medical image processing, 3D reconstruction and segmentation, 3D modeling of implant, and FDM printing of the designed implant. Medical imaging acquires images of bone defect regions using CT (Computed Tomography). The resulting CT scan produces detailed imaging of soft tissues, nerves and bone. For the current study, we obtained the CT scan dataset of the patient admitted to the Services Hospital, Lahore, Pakistan, for cranial surgery. The CT scan of the cranial region was recorded in DICOM (Digital Imaging and Communications in Medicine) file format, having a matrix size of 512 × 512 pixels.

**FIGURE 12:**
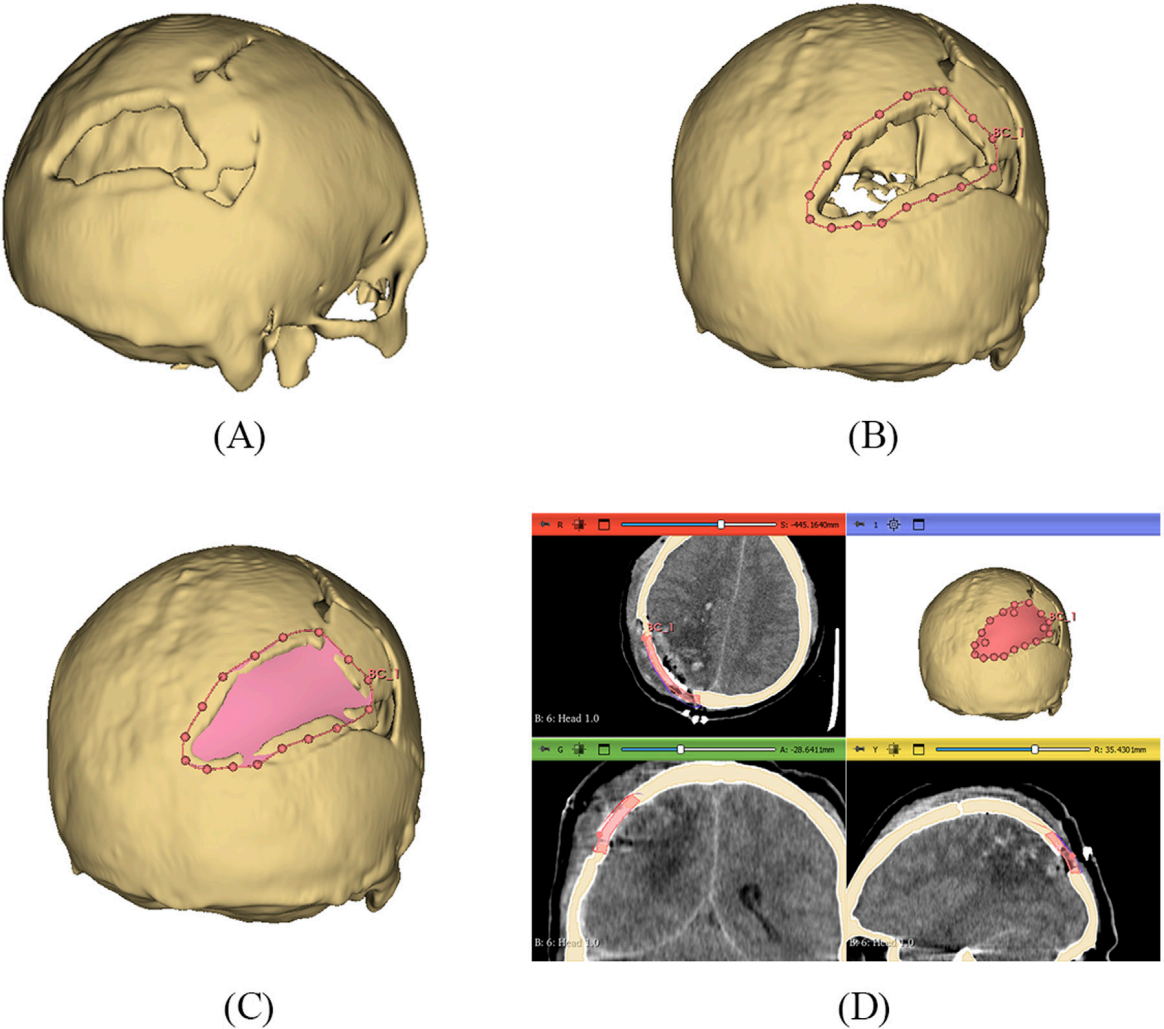
**(A)** 3D reconstructed skull model of patient **(B)** Contour points marking around defect **(C)** Planner surface patch generation **(D)** Thickness of 3D implant model.

The 3D slicer software was used to reconstruct the skull of the patient from two-dimensional CT scan images (Liu and Wang, 2024). The 3D reconstruction is a fundamental step that involves setting threshold values to remove noise and extract only the bone data, as depicted in Figure 12A. The threshold value is expressed in Hounsfield Unit (HU) and is used to isolate the bone from surrounding tissues and nerves. The minimum and the maximum HU values selected for bone intensity were 199 HU and 2906 HU, respectively, and the slider can be adjusted further to refine the selection. Finally, the segmented skull model can be used for further analysis and as a prototype for the next step, as it illustrates the shape and location of the cranial defect.

It is a typical case of the asymmetric cranial cavity as the defect lies at the back of the cranial and is not symmetric about the sagittal plane. Due to this asymmetric cavity, the baffle planner module under the ‘Slicer Heart’ extension is used to design the customized cranial. The contour points are marked manually by placing points along the boundary to generate a freeform surface patch around the defective region (Figures 12B, C).

**FIGURE 13:**
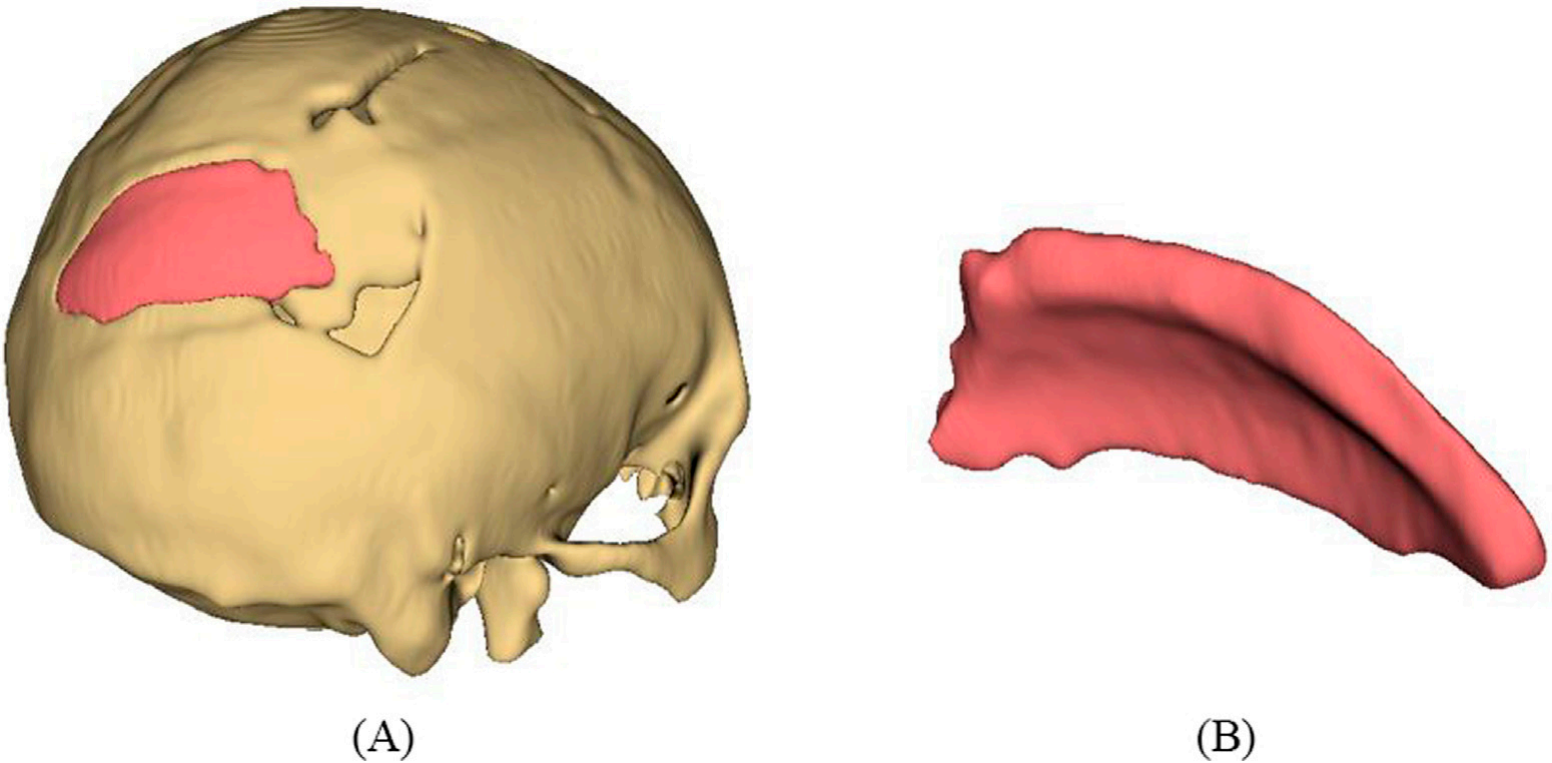
**(A)** Subtract Boolean operation and smoothing **(B)** Final cranial implant.

This surface patch creates a baffle model, and the curvature of this new model was adjusted by manually relocating the surface control points to create a more refined shape. By adjusting the contour points in axial, sagittal, and coronal views, the desired anatomical shape and curvature was achieved. The 3D solid was created by adjusting the scroll bar of skull thickness to 6 mm, as shown in Figure 12D. Now, in the ‘segment editor,’ the logical operator was used to subtract the 3D patch from the skull to remove the unwanted part of the implant, see Figure 13A. The segment boundaries were polished using the smoothing tool with a smooth factor of 1 mm. Finally, the shape of the cranial bone was restored, and the refined implant model was exported in STL (Standard Tessellation Language) file format. Figure 13B shows the final implant model.

**FIGURE 14:**
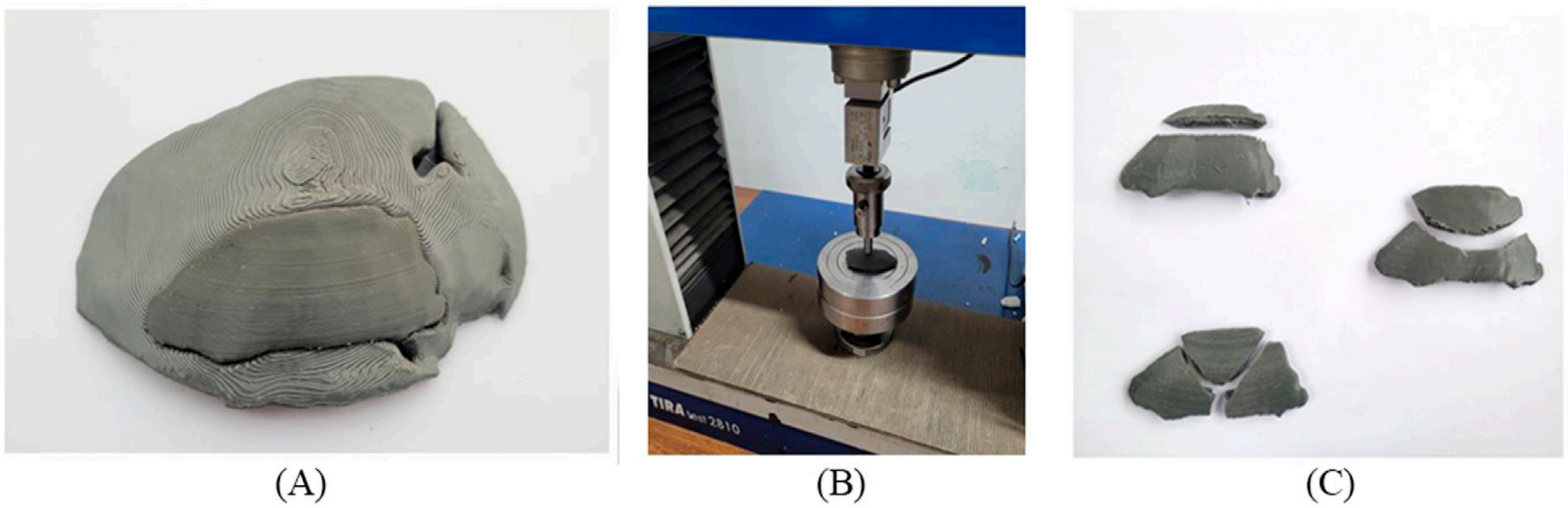
**(A)** Cranial bone model and fitting of the implant **(B)** Clamping tool and test setup **(C)** Fractured cranial implants after uniaxial load test.

The developed implant model was then 3D printed using PETG material. The printing parameters were set according to the results obtained by optimization to maximize mechanical strength. The result of mechanical strength has a direct relationship between laboratory mechanical tests and real-world medical applications to ensure that the implant can withstand forces exerted on the skull during normal activities. Figure 14A shows the physical mounting of the implant on the cranial bone model to check the visual alignment. The implant’s boundary perfectly matches the cranial bone boundary, which shows that the patient-specified implant is perfectly achieved with high dimensional accuracy. The customized implants perfectly align the patient’s unique cranial geometry, providing better mechanical support and enhancing the surgery’s overall performance. These customized implants allow better alignment with the patient’s unique cranial geometry, providing better mechanical support and enhancing the overall surgery performance. Minor changes in the geometry of the implant can be made based on the patient’s need before surgery.

It should be noted that the quality of the implant depends not only on the dimensional accuracy but also on its functioning. The fabricated implant was subjected to a uniaxial load for a compression test to evaluate its performance. The mechanical testing of PETG materials, including tensile strength and compressive strength, helps to determine whether the material can replicate the mechanical properties of natural bone. Depending on the loading direction, plenty of literature reported that the parietal bone’s average tensile strength came around 60–80 MPa and the compressive strength range around 150 MPa (EVANS and LISSNER, 1957; McElhaney et al., 1970). Figure 14B shows that the uniaxial compression test was carried out using a TIRA test machine with a load cell capacity of 10 kN. A hemispherical indenter of 10 mm diameter was used and the implant was statically loaded at a constant speed of 1 mm/sec. As reported by (Sharma et al., 2020), manufacturing a custom-shaped specimen holder can reflect the actual stress state. Instead of manufacturing a custom-shaped specimen holder, the implant was placed on a flat surface to have an operational judgment in this study. After the compression test, the broken implants are shown in Figure 14C. As a result of the experiment, the average peak force of the cranial implant was noticed at 1088 N. The maximum load a parietal bone can bear experimentally is 793.7 N (Motherway et al., 2009). Therefore, the maximum load the fabricated implant can bear seems to align with the parietal bone data, making it suitable for small-size defects and repairs. Mechanical testing under compressive load provides insight into the durability of an implant under basic mechanical stresses from daily activities such as head impacts or repeated loading.

## 5 Conclusion

In this work, the cranial implant was developed using patient CT scan data, and mechanical strength of PETG material was examined by optimizing the FDM printing parameters. In this study, the influence of layer height (0.1 mm, 0.2 mm, 0.3 mm), line width (0.4 mm, 0.6 mm, 0.8 mm), and print speed (25 mm/s, 35 mm/s, 45 mm/s) on the tensile and compressive strength was explored. Line width seems to be an essential parameter; the modification in line width, rather than setting it to default, can help achieve better mechanical strength. The mechanical strength outcomes provide helpful information about using PETG material in medical applications. From the findings, the following conclusion can be drawn.

1) According to ANOVA results, layer height exhibits a more significant influence on tensile strength. Additionally, the interaction between layer height (A) and line width (B) contributes substantially compared to the interaction of line width and print speed for the tensile strength of PETG. Regarding contributions to the total sum of squares (SS), percentages for linear, interactions and quadratics effects are 62.4%, 4.32% and 32.3%, respectively. The reduction in layer height and printing speed values tends to increase the tensile strength. Regarding line width, the tensile strength initially increases and then gradually declines, with a maximum value of 51.6 MPa observed near 0.6 mm.
2) As the line width increases, the compressive strength increases due to reduced voids. The interaction effect between layer height and print speed is less significant than the interaction between layer height and line width, contributing significantly to compressive strength. By decreasing the value of layer height and printing speed, a substantial increase in compressive strength was observed.
3) For both tensile and compressive strength, the relationship between the experimental results and predicted data represents the significance of the model. The *R* ^2^, predicted *R* ^2^ and adjusted *R* ^2^ values are close to 1, which indicates the model’s accuracy according to the current data.
4) The validation analysis was conducted using the optimized parameters of layer height 0.12 mm, line width 0.77 mm and print speed 25.75 mm/s. Less than 2% error was observed between the experimental and predicted results.
5) The results presented in this paper are beneficial in identifying the limitations of the default value of line width and help achieve the desired values of mechanical strength. It is observed that the line width and layer heights significantly increased the mechanical strength of the part. It should also be noted that tensile and compressive strength have been analyzed, and the value of the ultimate tensile stress is lower than that of the compressive stress of the printed samples. This comparison of results helps to make better products using FDM.
6) The framework for the PSI design was presented by utilizing the baffle planner tool to develop the 3D cranial implant model. Manual adjustments of thickness and refinement of contours are required, so the process relies on the experience of the user. The implant was then 3D printed for PETG against the optimized parameter, and the physical fitting of the implant and skull model was found satisfactory. The implant was then tested mechanically using a uniaxial compressive load, and the average maximum load was about 1088 N.
7) One of the major limitations of PETG implants is their lower mechanical strength compared to that of metallic or ceramic material, which limits their effectiveness in load-bearing applications and may fail under high or sudden forces. PETG seems to be an acceptable material for cranial bone reconstruction, especially for smaller defects that do not require high load capacity. Further investigations should be conducted to explore the use of PETG in clinical trials. Further research should be conducted for *in-vivo* and post-surgery evaluation of the PETG implants, focusing on animal studies and clinical trials, which will help further correlate laboratory results with clinical practice.

## Data Availability

The original contributions presented in the study are included in the article/supplementary material, further inquiries can be directed to the corresponding author.
